# Identification of three cultivated varieties of *Scutellaria baicalensis* using the complete chloroplast genome as a super-barcode

**DOI:** 10.1038/s41598-023-32493-9

**Published:** 2023-04-05

**Authors:** Yuan Jiang, Chenghao Zhu, Shangtao Wang, Fusheng Wang, Zhirong Sun

**Affiliations:** 1 School of Chinese Materia Medica, Beijing University of Chinese Medicine, Beijing, China; 2 Dingxi Academy of Agricultural Sciences, Dingxi, China

**Keywords:** Phylogenetics, Genetic markers, Genome, Genomics

## Abstract

*Scutellaria baicalensis* has been one of the most commonly used traditional Chinese medicinal plants in China for more than 2000 years. The three new varieties cultivated could not be distinguished by morphology before flowering. It will hinder the promotion of later varieties. Chloroplast DNA has been widely used in species identification. Moreover, previous studies have shown that complete chloroplast genome sequences have been suggested as super barcodes for identifying plants. Therefore, we sequenced and annotated the complete chloroplast genomes of three cultivated varieties. The chloroplast genomes of SBW, SBR, and SBP were 151,702 bp, 151,799 bp, and 151,876 bp, which contained 85 protein-coding genes, 36 tRNA genes, and 8 rRNA genes. The analysis of the repeat sequences, codon usage, and comparison of chloroplast genomes shared a high degree of conservation. However, the sliding window results show significant differences among the three cultivated varieties in *mat*K-*rps*16 and *pet*A-*psb*J. And we found that the *mat*K-*rps*16 sequence can be used as a barcode for the identification of three varieties. In addition, the complete chloroplast genome contains more variations and can be used as a super-barcode to identify these three cultivated varieties. Based on the protein-coding genes, the phylogenetic tree demonstrated that SBP was more closely related to SBW, in the three cultivated varieties. Interestingly, we found that *S. baicalensis* and *S. rehderiana* are closely related, which provides new ideas for the development of *S. baicalensis*. The divergence time analysis showed that the three cultivated varieties diverged at about 0.10 Mya. Overall, this study showed that the complete chloroplast genome could be used as a super-barcode to identify three cultivated varieties of *S. baicalensis* and provide biological information, and it also contributes to bioprospecting.

## Introduction

*Scutellaria baicalensis* Georgi (*S. baicalensis*) is a traditional Chinese medicinal plant that belongs to the family Lamiaceae. As one of the most commonly used Chinese medicinal materials in China, it has been used as medicinal material for more than 2000 years since being first recorded in the Shen-nong-ben-cao-jing (The Classic of Herbal Medicine)^1^. To date, it has been included in over 90% of TCM formulas for treating colds^2^. Flavonoids and their glycosides are the main bioactive compounds of *S. baicalensis*. The main components of root-specific 40 deoxygenated flavonoids are baicalein, baicalin, wogonin, and carboxylase A^3–5^. According to current pharmaceutical investigations, *S. baicalensis* active compounds exhibit significant pharmacological actions such anti-oxidation, anti-bacterial, anti-viral, anti-tumor, and anti-inflammation^6–8^. And it has been widely used for the treatment of various diseases such as pneumonia, diarrhea, infections, colitis, and hepatitis^9–13^.

**Figure 1:**
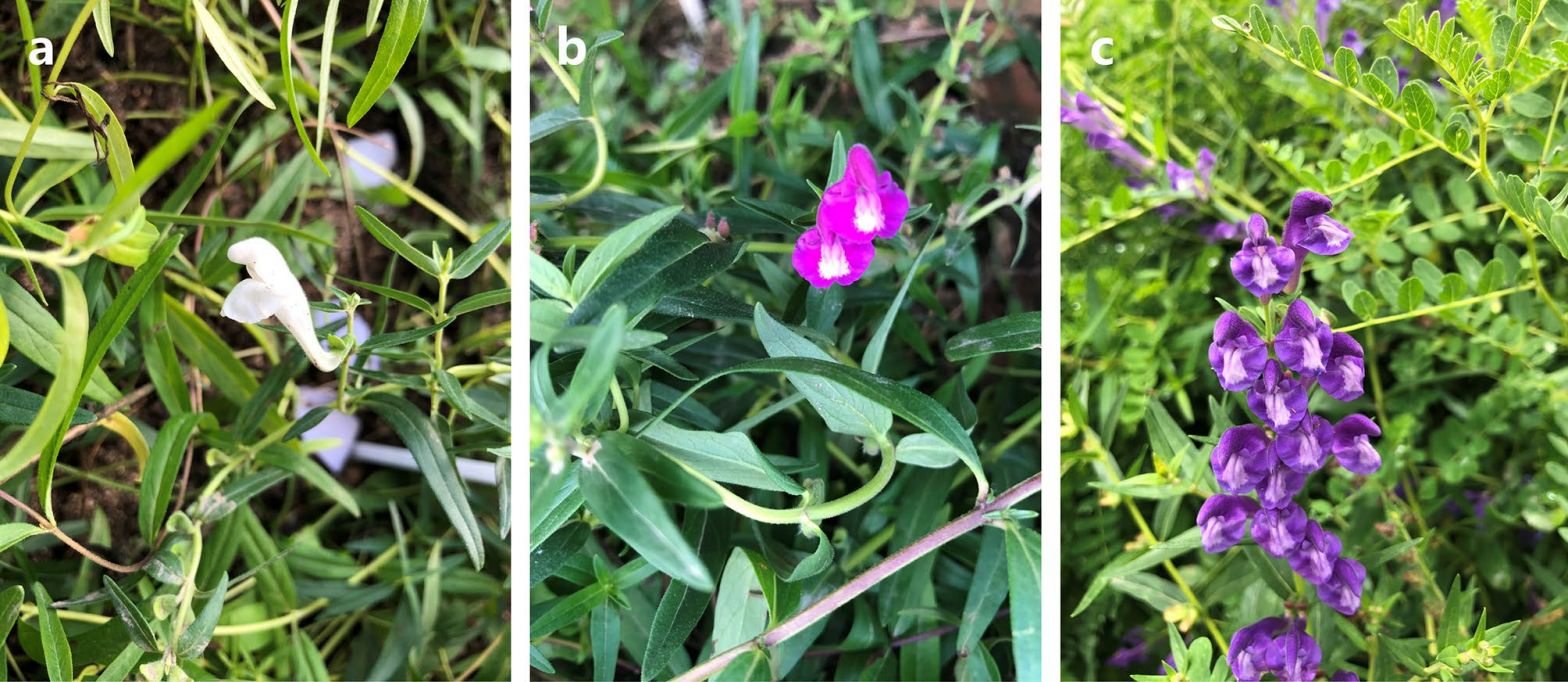
The phenotype of three cultivated varieties (**a**: SBW, **b**:SBR, **c**: SBP).

Recently, the Corona Virus Disease 2019 (COVID-19) spread worldwide quickly^14^. It is recently found that *S. baicalensis* has significant curative effects on the treatment of COVID-19^15,16^. It has led to the large-scale cultivation of *S. baicalensis* in China, and researchers are also actively cultivating new varieties. This study used wild *S. baicalensis* resources in Dingxi City as raw materials. The single plant optimization method screened varieties with plant agronomic characters, susceptibility, and drug grade indicators. Then we screened three new cultivated varieties of *S. baicalensis* with high quality and yield through strain identification, strain comparison, multi-point test, and regional production test. But it is not clear which varieties can be widely used. The morphological differences between the three cultivated varieties are mainly due to the difference in flower color (Fig. 1), namely white (SBW), rose (SBR), and purple (SBP). However, it was impossible to distinguish the three cultivated varieties before flowering. In addition, *S. baicalensis* typically has purple flowers, but a rare white or rose flower phenotype has been cultivated, showing great ornamental potential. Accurate identification of varieties also provides a basis for homozygous breeding.

The chloroplast (cp) is an essential organelle that plays a crucial role in plant photosynthesis and several critical biochemical processes^17^. Due to its slow mutation rate, abundance within plants, relatively small genome size, and haploid inheritance^18^. The cp DNA has been widely used in many research fields, such as taxonomic revision, systematic evolution, and species identification^19,20^. Moreover, previous studies have shown that complete cp genome sequences have been suggested as super barcodes for identification of plants^21,22^.

The complete cp genomes of three cultivated varieties were sequenced and annotated in this study. To determine the internal differences, we examined the general characteristics and compared the sequence differences. Moreover, we explored the phylogenetic position to decipher the genetic relationship amongst the three cultivated varieties to provide the basis for the variety breeding. The result of this study will provide abundant genetic information on *S. baicalensis*, and serve as the theoretical basis for expanding its medicinal resources.

## Materials and methods

### Plant materials

Fresh, healthy leaf tissues of three cultivated varieties of *S. baicalensis* (SBW, SBR, and SBP) were collected from the Germplasm Resources Nursery of Dingxi Academy of Agricultural Sciences (Dingxi, China, 35°6′38″N, 118°21′48″E) (Fig. 1). The specimens were identified by Professor Zhirong Sun following the taxonomic key and external morphology diagnosis proposed by Flora Reipublicae Popularis Sinieae. The voucher specimens were deposited at the herbal medicine library of the school of Chinese materia medica, Beijing University of Chinese medicine.

### DNA extraction and sequencing

The fresh leave of three cultivated varieties was frozen in liquid nitrogen and stored in a − 80 °C refrigerator for DNA extraction. DNA extraction was performed using Plant Genomic DNA Kit (Tiangen Biotech, Beijing) following the manufacturer instructions. Around 20–30 mg of dried tissue or 50–60 mg of frozen tissue was used in each extraction. After DNA isolation, 1 μg of purified DNA was fragmented and used to construct short-insert libraries (insert size 300–500 bp) according to the manufacturer’s instructions (Illumina HiSeq X-Ten) for sequencing.

### Cp genome assembly and annotation

The high-quality reads were assembled using GetOrganelle v.4.0 and then annotated by CpGAVAS2^23^. The annotations of tRNA genes were confirmed by using tRNAscan-SE v.2.03^24^. The Bowtie2 and SAMtools were used to perform mapping the reading to the assembled genome, and evaluate the effectiveness of the assembly results. The cp genomes of SBP, SBR, and SBW were submitted to GenBank at the National Center of Biotechnology Information (NCBI), and the accession numbers were OP837955, OP837956, and OP837957, respectively. Fully annotated plastome circular diagrams were drawn by a website (https://irscope.shinyapps.io/Chloroplot/).

### Codon usage

The protein-coding genes were extracted by Phylosuite v.1.2.2^25^. Relative synonymous codon usage (RSCU) and codon usage values were analyzed by CodonW v.1.4.2. Moreover, the RSCU values were shown in a heatmap by TBtools^26^.

### Repeat analysis and comparative analyses

Repetitive sequence analyses were performed using CPGAVAS2 analysis. Tandem repeats were identified using default settings by Tandem Repeats Finder^27^. The Misa.pl was used to screen the simple sequence repeats (SSRs)^28^. The scattered repetitive sequences were found by using VMATCH. The REPuter was used to determine the size and location of the oligonucleotide repeats (ORs)^29^. The complete cp genomes of three cultivated varieties were compared by mVISTA^30^, and the genome of *S. baicalensis* (NC027262) was used as the reference sequence for annotation. Sliding window analysis was conducted to assess the nucleotide diversity (Pi) values of the cp genomes by DnaSP v6 (window length = 300 bp, step size = 25 bp). IRscope^31^ was used to analyze inverted repeated traction and expansion at cp genomes’ junctions.

### Identification and validation of barcode for species discrimination

According to the results of DNAsp, we chose the high variation region to distinguish the three cultivated varieties. Primers to discriminate between the three cultivated varieties under study were designed on the variable intergenic regions using Snapgene 6.2.1 (Snapgene from Insightful Science, available at http://www.snapgene.com, last used in 2023). PCR amplifications were performed in a final volume of 20 μL with 10 μL 2 × Taq PCR Master Mix, 0. 5 μM of each primer, 5 μL template DNA, and 4 μL ddH_2_O following the manufacturer’s instructions (Mei5 Biotechnology, Co., Ltd). All amplifications were carried out in a Pro-Flex PCR system (Applied Biosystems, Waltham, MA, USA) under the following conditions: denaturation at 95 °C for 3 min, followed by 36 cycles of 94 °C for 25 s and 55 °C for 10 s, and 72 °C for 2 min as the final extension following the manufacturer’s instructions (Mei5 Biotechnology, Co., Ltd). PCR amplicons were visualized on 1% agarose gels, purified and then subjected to bidirectional Sanger sequencing on an ABI 3730 XL instrument (Applied Biosystems, USA) using the same set of primers used for PCR amplification with BigDye v3.1 chemistry (Applied Biosystems) following manufacturer’s instructions. All amplifications were repeated twice for each variety.

### Phylogenetic analysis and divergence times analysis

Phylogenetic analysis was performed based on 21 complete cp genomes, including the three assembled sequences in our study, 16 cp genomes downloaded from the NCBI (12 *Scutellaria*, 1 *Pogostemon*, 1 *Ajuga*, 1 *Lavandula,* and 1 *Ocimum*), and *Tulipa gesneriana* (NC063831) and *Aloe vera* (NC035506) as outgroup. A total of 86 shared protein-coding genes were extracted and then concatenated and aligned using MAFFT v7.307^32^. Subsequently, the alignment was conducted based on Bayesian inference (BI) in MrBayes using the GTR + I + G evolution model^33^. The parameter was set to run for five million generations and sampled every 1000 generations, with all other settings left at their defaults, and the first 25% of each run was discarded as burn-in. The alignment was also evaluated using bootstrap analysis on 1000 in a maximum likelihood (ML) by RAxML^34^, with parameters: raxmlHPC-PTHREADS-SSE3 -fa -N 1000 -m GTRGAMMA- x551,314,260 -p 551,314,260 -o Fritillaria_cirrhosa_NC_024728, Fritillaria_thunbergii_NC_034368 -T 20, 1000 replications and best-fit model selection. Besides, Modeltest was used to determine the most appropriate model of DNA sequence evolution for the combined 87-gene dataset. Moreover, MrBayes was run for 5,000,000 generations, sampling, and printing every 500. Two independent MCMC runs using four chains (with the default heating schedule) were conducted per Bayesian analysis. Branch support was calculated from the posterior distribution of Bayesian trees after discarding the first 25% of the trees as burn-in and 1000 ML bootstrap pseudoreplicates.

We used the software MEGA^35^ for molecular clock analysis on the shared cp protein-coding genes alignment, using fossil information of *Arabidopsis thaliana* (53–82 million years ago, Mya), *Oryza sativa* (148–173 Mya), and the family Labiatae (49 Mya)^36–38^. Moreover, another molecular clock tree was constructed based on an ML tree using BEAST^39^. Phylogenetic inference following MCMC analysis with default settings was performed (20,000,000 generations, Yule speciation tree prior to the substitution rate, the trees sampled every 1000 generations) under a strict clock approach. TRACER software was used to check the acceptability and convergence to the stationary distribution of trees^40^, while TREEANNOTATOR software was used to generate the maximum clade credibility tree from the obtained trees after setting a burning-in of 10%^41^. The tree was visualized with FigTree (v. 1.4.4; http://tree.bio.ed.ac.uk/software/figtree/).

## Results and discussion

### Characteristics of three cultivated varieties

**Figure 2:**
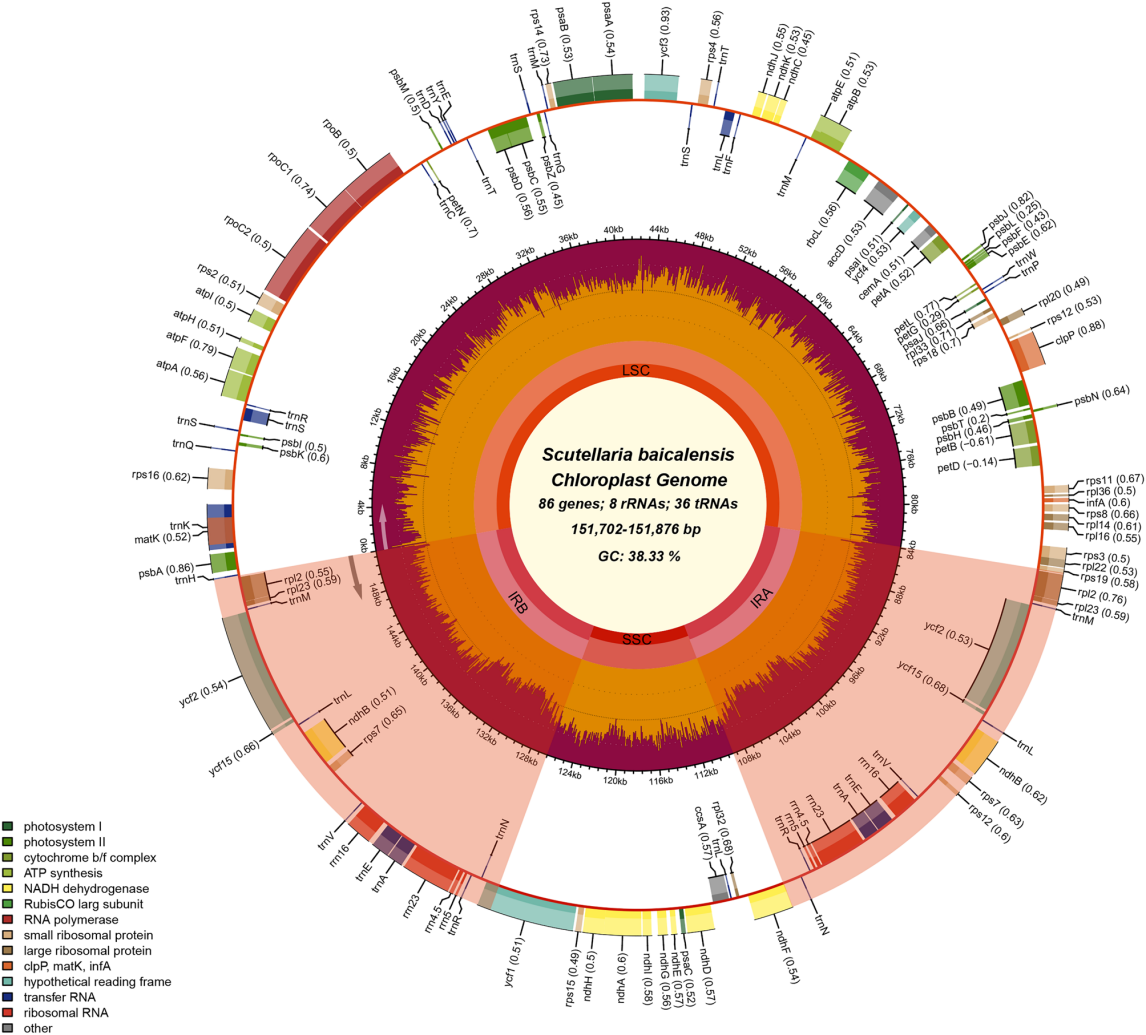
Cp genome map of three cultivated varieties. Genes lying outside the circle are transcribed in the clockwise direction, while those insides are transcribed in the counterclockwise direction. The colored bars indicate different functional groups. The darker red area in the inner circle denotes GC content, while the orange corresponds to the AT content of the genome. LSC: large single copy, SSC: small single copy, IRA/B: inverted repeat.

The coverage of three cultivated varieties of cp genomes was even and not zero (Fig. S1). The results indicated that the cp genome splicing results of the three cultivated varieties were correct and there was no heteroplasmy. The size and content of these genomes have been analyzed (Table 1). The cp genome size of SBW (151,702 bp) was the most minor, and SBP (151,876 bp) was the largest. All three cultivars cp genomes of *Scutellaria* exhibited a typical quadripartite structure (Fig. 2), with two inverse-repeat (IR, including IRa and IRb, 25,261–25,265 bp) regions separated by large single-copy (LSC, 83,878–84,025 bp) and small single-copy (SSC, 17,294–17,330 bp) regions. These cp genomes exhibited identical gene content and type and were generally classified into self-replication, photosynthesis, and other genes (Table 2). A total of 129 genes in these species, including 85 protein-coding genes, 36 tRNA genes, and 8 rRNA genes. The results were identical to the other members of the genus *Scutellaria*. Compared with most angiosperms, the *psb*H, *rpo*A, *chI*B, *chI*L, and *ycf*68 were lost during evolution. The total GC content of three cultivars cp genomes was 38.33% but was unevenly distributed in each region (Table 1). The GC content in IR region (43.61%) was higher than LSC (36.32–36.33%) and SSC (32.61–32.66%). However, the GC content was lower than AT content. These results agree with previous studies of angiosperms, such as the genus of *Polygonatum* and *Epimedium*^42,43^. The circular map of cp genomes was provided for three cultivars in Fig. 2.

**Table 1:** Summary of the cp genome features for the three cultivated varieties.

Cultivar	Region	Length (bp)	GC content (%)
SBW	Total	151,702	38.33
	IR	25,265	43.61
	SSC	17,294	32.61
	LSC	83,878	36.33
SBR	Total	151,799	38.33
	IR	25,263	43.61
	SSC	17,330	32.66
	LSC	83,943	36.33
SBP	Total	151,876	38.33
	IR	25,261	43.61
	SSC	17,329	32.65
	LSC	84,025	36.32

LSC: large single-copy region; SSC: small single-copy region; IR: inverted repeat.

**Table 2:** List of genes present in the cp genome of the three cultivars of *Scutellaria*.

Category of genes	Group of genes	Name of genes	Number
rRNA	rRNA	*rrn*16S (×2), *rrn*23S (×2), *rrn*5S (×2), *rrn*4.5S (×2)	8
tRNA	tRNA	*trnA-UGC (×2), *trn*C-GCA, *trn*D-GUC, *trnE-UUC (×3), *trn*F-GAA, *trn*G-GCC, *trn*H-GUG, *trnK-UUU, *trn*L-CAA (×2), *trnL-UAA, *trn*L-UAG, *trn*M-CAU (×2), *trn*M-CAU (×2), *trn*N-GUU (×2), *trn*P-UGG, *trn*Q-UUG, *trnR-ACG* (×2), *trnR-UCU*, *trnS-CGA, *trn*S-GCU, *trn*S-GGA, *trn*S-UGA, *trn*T-GGU, *trn*T-UGU, *trn*V-GAC (×2), *trn*W-CCA, *trn*Y-GUA	36
Genes for photosynthesis	Subunits of ATP synthase	*atp*A, *atp*B, *atp*E, *atpF, *atp*H, *atp*I	6
	Subunits of photosystem II	*psb*A, *psb*B, *psb*C, *psb*D, *psb*E, *psb*F, *psb*I, *psb*J, *psb*K, *psb*L, *psb*M, *psb*N, *psb*T, *psb*Z, **ycf3	15
	Subunits of NADH-dehydrogenase	*ndhA, *ndhB (×2), *ndh*C, *ndh*D, *ndh*E, *ndh*F, *ndh*G, *ndh*H, *ndh*I, *ndh*J, *ndh*K	12
	Subunits of cytochrome b/f complex	*pet*A, *petB, *petD, *pet*G, *pet*L, *pet*N	6
	Subunits of photosystem I	*psa*A, *psa*B, *psa*C, *psa*I, *psa*J	5
	Subunit of rubisco	*rbc*L	1
Self replication	Large subunit of ribosome	*rpl*14, *rpl16, *rpl2 (×2), *rpl*20, *rpl*22, *rpl*23 (×2), *rpl*32, *rpl*33, *rpl*36	11
	DNA dependent RNA polymerase	*rpo*B, *rpoC1, *rpo*C2	3
	Small subunit of ribosome	*rps*11, *rps*12 (×2), *rps*14, *rps*15, *rps16, *rps*18, *rps*19, *rps*2, *rps*3, *rps*4, *rps*7 (×2), *rps*8	14
Other genes	Subunit of Acetyl-CoA-carboxylase	*acc*D	1
	c-type cytochrom synthesis gene	*ccs*A	1
	Envelop membrane protein	*cem*A	1
	Protease	**clpP	1
	Translational initiation factor	*inf*A	1
	Maturase	*mat*K	1
Unkown	Conserved open reading frames	*ycf*1, *ycf*15 (×2), *ycf*2 (×2), *ycf*4	6

*Gene with one intron; **gene with two introns.

Additionally, the number and types of introns were similar among the cultivars of *S. baicalensis*, except for SBW, there is no intron in *rpl16*. Eighteen genes each contained one intron, including *rpl*2 (× 2), *ndh*B (× 2), *trn*E-UUC (× 2), *trn*A-UGC (× 2) were located in the IR, and the genes (*trn*K-UUU, *rps*16, *trn*S-CGA, *atp*F, *rpo*C1, *trn*L-UAA, *pet*B, *pet*D, and *rpl*16) were located in the LSC, and the *ndh*A was the only present in the SSC region. In addition, the *ycf*3 and *clp*P comprise two introns (Table S1). According to the statistics of intron length, *trn*K-UUU gene has the longest intron in the cp genome of the three cultivated varieties, which is also found in *Atractylodes*^44^. In addition, the *mat*K gene was located within the intron of the *trn*K-UUU gene, which putatively codes for a plastid intron maturase^45,46^.

### Codon usage

**Figure 3:**
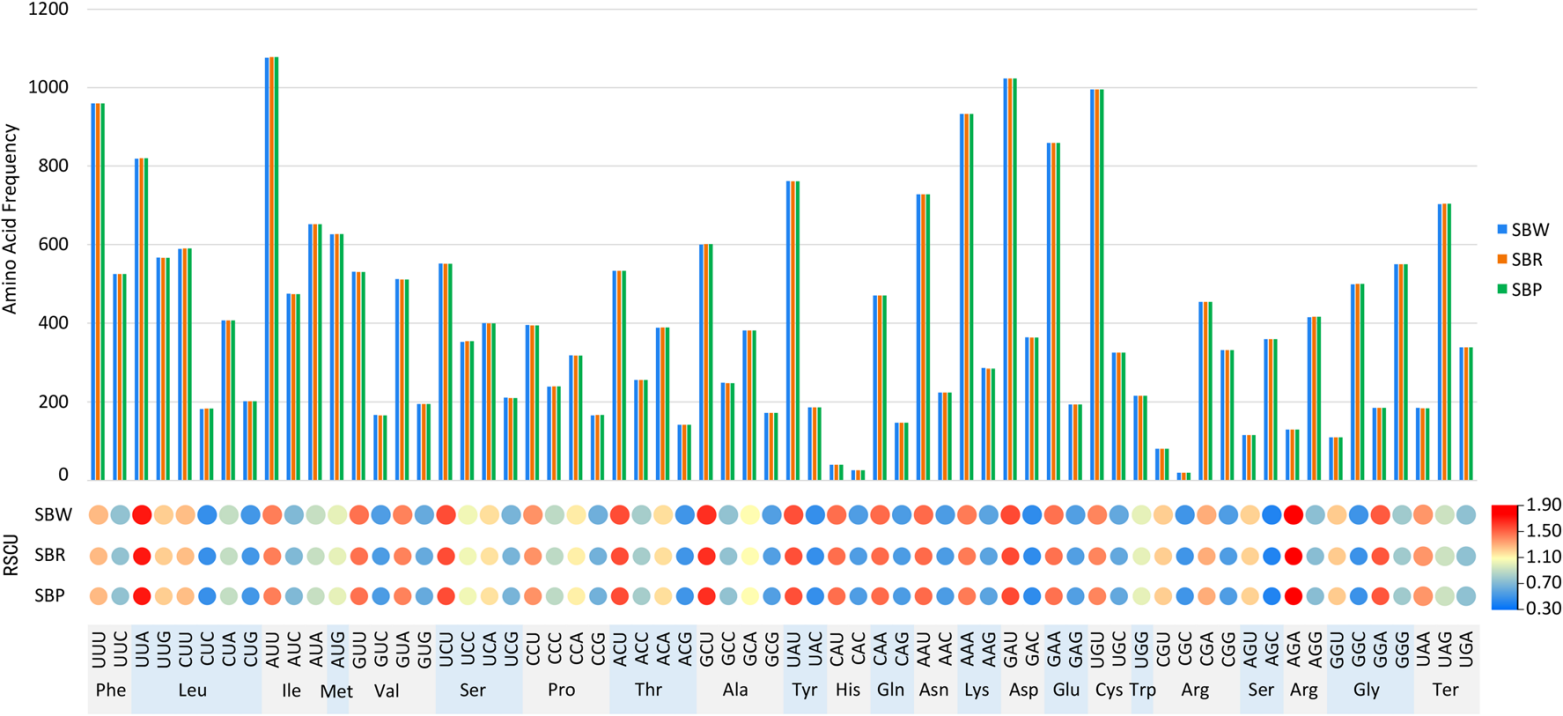
The RSCU values of all protein-coding genes for three cultivated varieties. Color key: the red values indicate higher RSCU values, and the blue values indicate lower RSCU values.

The cp genome of three cultivated varieties of *S. baicalensis* contained 64 codons encoding 20 amino acids. The result of the RSCU revealed that 31 codons were used frequently in these cultivars, with the highest frequency of AGA followed by UUA (Fig. 3). Moreover, the codon exhibited a strong bias toward an A or T at the third position. The codons that contain A/T at the 3′ end mostly have RSCU ≥ 1, whereas the codons are having C or G at the 3′ end mostly have RSCU ≤ 1. Amino acid frequency analyses revealed the highest frequency of Leucine and Iso-leucine, whereas Tryptophane was a rare amino acid. In general, we found high similarities in codon usage and amino acid frequency among the three cultivated varieties, and both contain high AT content. Similar results were found in the cp genome of other angiosperms^47,48^.

### Repeat analysis

**Figure 4:**
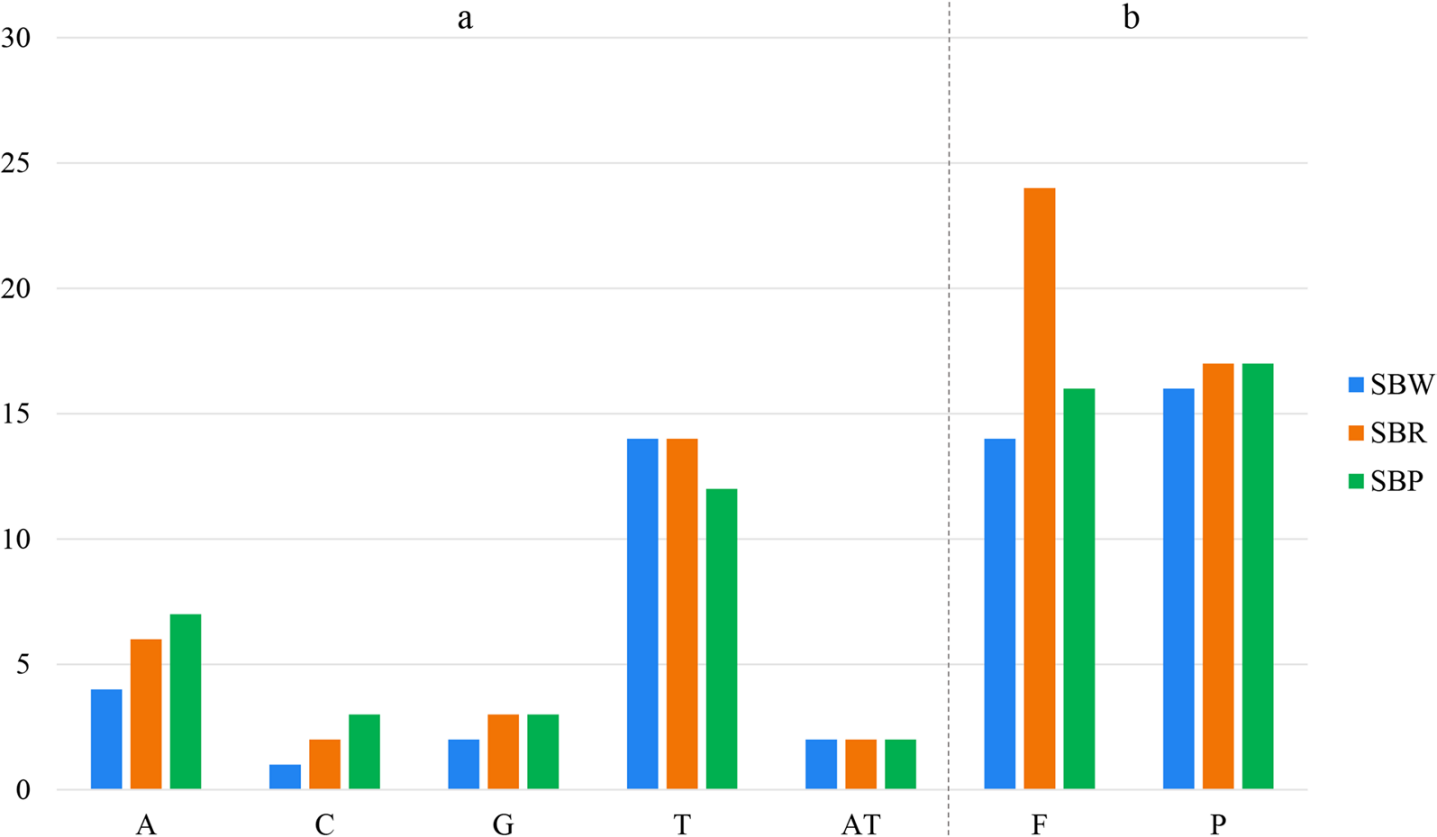
Comparison of repeats in three cultivated varieties. (**a**) SSR distributed situation in the cp genomes of five species. (**b**) Long repeats classification of five species. F—forward repeats; P—palindromic repeats.

Our analyses identified SSRs per genome composed of mono- to di- nucleotide repeating units (Fig. 4a). The number and type of SSRs in SBR and SBP were similar, with 25 single nucleotide repeats and 2 dinucleotide repeats. SBW contains only 21 single nucleotide repeats less than SBR and SBP. Moreover, in three cultivated varieties, the main type of mononucleotide repeats was T. Oligonucleotide repeats analyses by REPuter detected two types of repeats: Forward (F) and Palindromic (P). Figure 4b showed that the number of repeats varied in three cultivated varieties. We discovered that 30 repeats in SBW include 14 forward and 16 palindromic, 41 repeats in SBR include 24 forward and 17 palindromic, and 33 repeats in SBP include 16 forward and 17 palindromic. Most of the repeats ranged in size from 30 to 40 bp in three cultivated varieties. This result showed that SBW and SBP were more similar than SBR. We also evaluated the number of repeats about the species' phylogenetic position using the topology in Fig. 8. The results confirmed the random distribution of repeat numbers independent of phylogenetic position.

### Comparative cp genomic analysis

**Figure 5:**
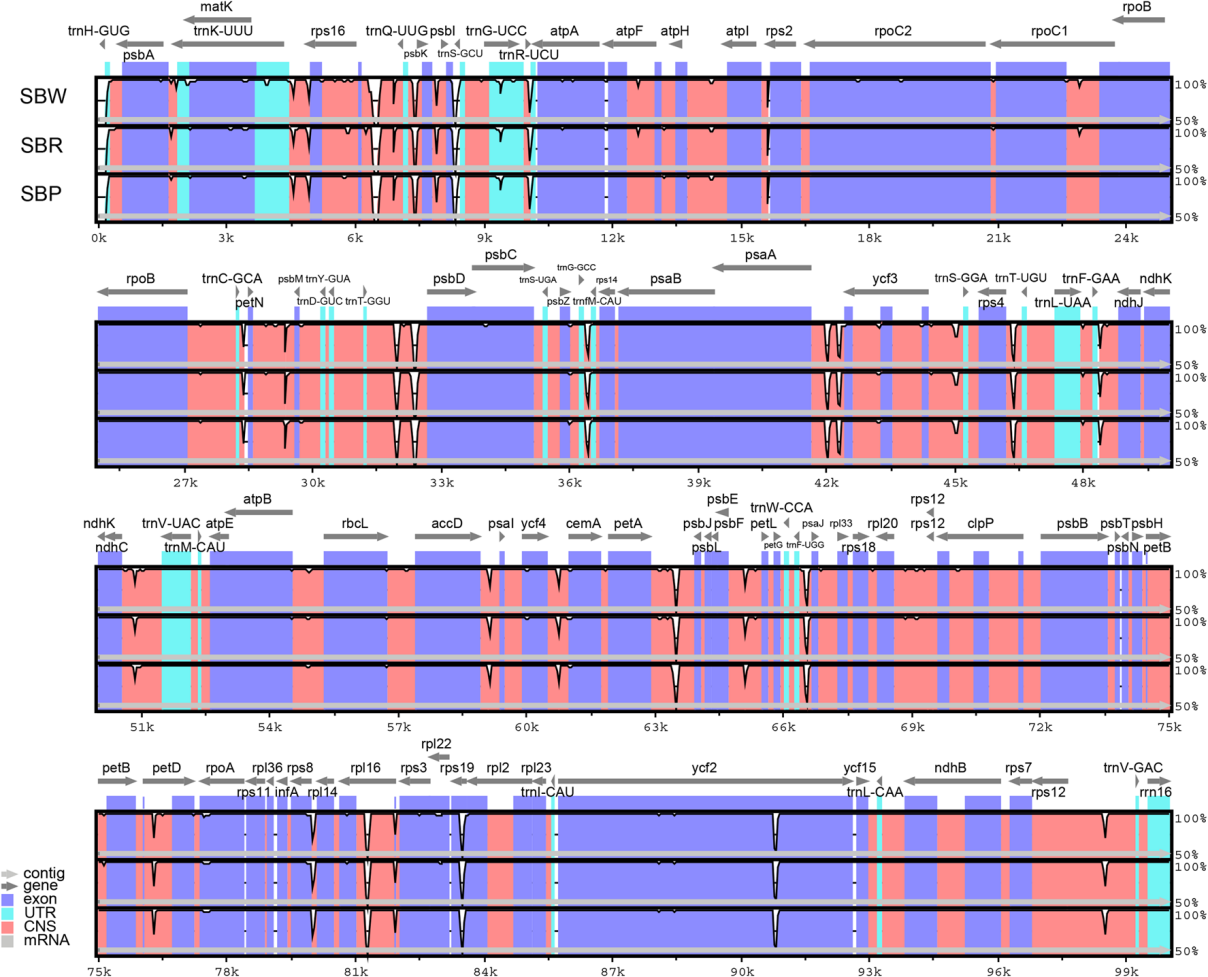
Comparison of three cultivated varieties cp genomes using *S. baicalensis* (NC027262) annotation as a reference. The vertical scale indicates the percentage of identity, ranging from 50 to 100%. The horizontal axis shows the coordinates within the cp genome. Genome regions are color-coded as exons, introns, and intergenic spacer (IGS), and the Gray arrows indicate the direction of transcription of each gene. Annotated genes are displayed along the top.

The cp genomes of the three cultivated varieties were compared by mVISTA^30^, and the *S. baicalensis* (NC027262) was used as the reference sequence for annotation. The Fig. 5 showed that the three cultivated varieties exhibit similar variation sites and degrees of variation. The coding regions (CDS) were more conserved than the intergenic spacers (IGS). The high divergence in IGS were found in *rps*16-*trn*Q(UUG), *trn*Q(UUG)-*psb*K, *psb*L-*trn*S(GCU), *trn*R(UCU)-*atp*A, *trn*T(GGU)-*psb*D, *trn*G(GCC)-*trnf*M(CAU), *psa*A-*ycf*3, *rps*4-*trn*T(UGU), *pet*A-*psb*J, *trn*F(UGG)-*psa*J. Furthermore, some mutations of CDS were found in *rps*19, *rpl*16, *ycf*2. These high variation region sequences could be used to distinguish wild species from cultivated species. Moreover, the result showed that IR regions had lower sequence divergence than LSC and SSC regions.

**Figure 6:**
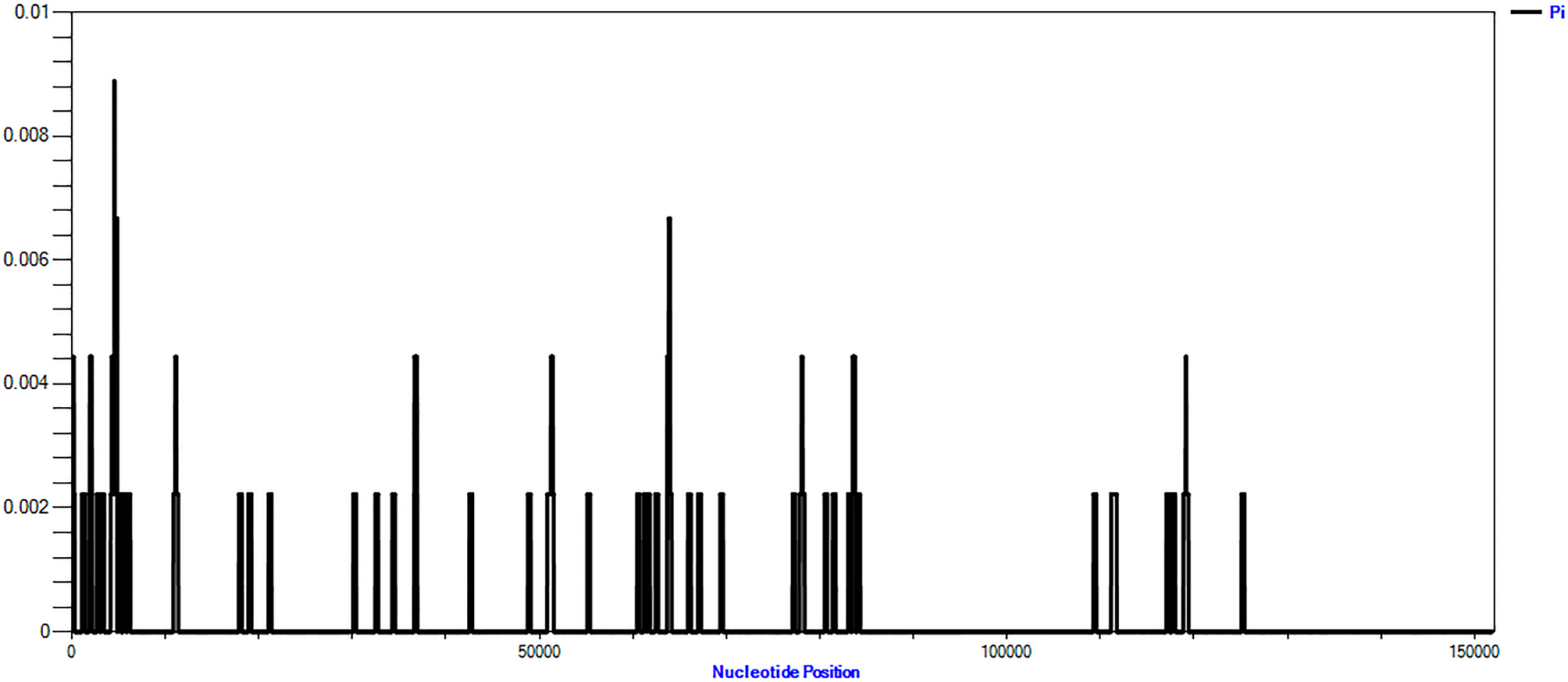
Sliding window analysis of the entire cp genome of three cultivated varieties (window length: 300 bp; step size: 25 bp). X-axis: position of the window; Y-axis: nucleotide diversity of each window.

In order to explore the sequence divergence between the three cultivated varieties, nucleotide diversity (Pi) was estimated to indicate the variability of potential plastid regions. The values of Pi ranged from 0 to 0.01 (Fig. 6). Among them, 4427–5018 bp region showed high nucleotide diversity (Pi: 0.0067–0.0089). This region was identified as an IGS in *mat*K-*rps*16. Besides, another high variable region (Pi: 0.0067) appears at 63,718–64,092 bp, located at *pet*A-*psb*J. Therefore, the complete cp genome could be used as a super-barcode to identify the three cultivated varieties.

### Inverted repeats contraction and expansion

**Figure 7:**
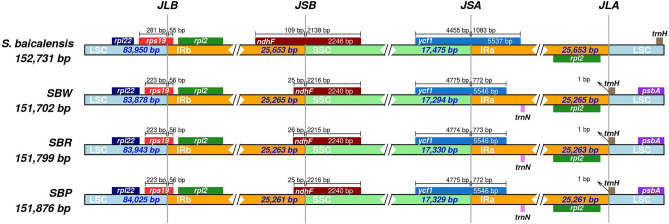
Comparison of quadripartite junction sites in three cultivated varieties cp genomes. Gene transcribed clockwise are presented below the track, whereas transcribed counterclockwise are presented on top of the track. The start and end of each gene from the junctions have been shown with arrows. The T scale bar above or below the track shows genes integrated from one region of the cp to another. JLB (IRb/LSC), JSA (SSC/IRa), JSB (IRb/SSC), and JLA (IRa/LSC) denotes the junction sites between the quadripartite regions of the genome.

The inverted repeats contraction and expansion revealed variation at LSC/IRs/SSC junctions. The types of junctions in three cultivated varieties and *S. baicalensis* (NC027262) were different (Fig. 7). In all species, a truncated copy of the *rps*19 gene was found at the IRb/LSC junction; the *rpl*22 gene was found entirely in the LSC region; and the *rpl*2 gene was found entirely in the IRb region. Another truncated copy of *ndh*F gene was found at the junction of IRb/SSC in all species, which starts in IRb regions and integrates into the SSC region. Interestingly, compared with the three cultivated varieties, the *ndh*F gene of *S. baicalensis* was longer in IRb. Moreover, a truncated copy of *ycf*1 was found in SSC/IRa junction, which was longer in IRa of *S. baicalensis*. In three cultivated varieties, *trn*N was observed to present entirely in the IRa region, and the *trn*H was completely exists in LSC and only one bp from the junction of IRa/LSC. In comparison, the *trn*H gene of *S. baicalensis* was 178 bp from the junction of IRa/LSC. These results show that the cp genome of three cultivated varieties displays a unique IR contraction compared to the wild species.

### Specific DNA barcode maker design for three cultivated varieties

**Figure 8:**
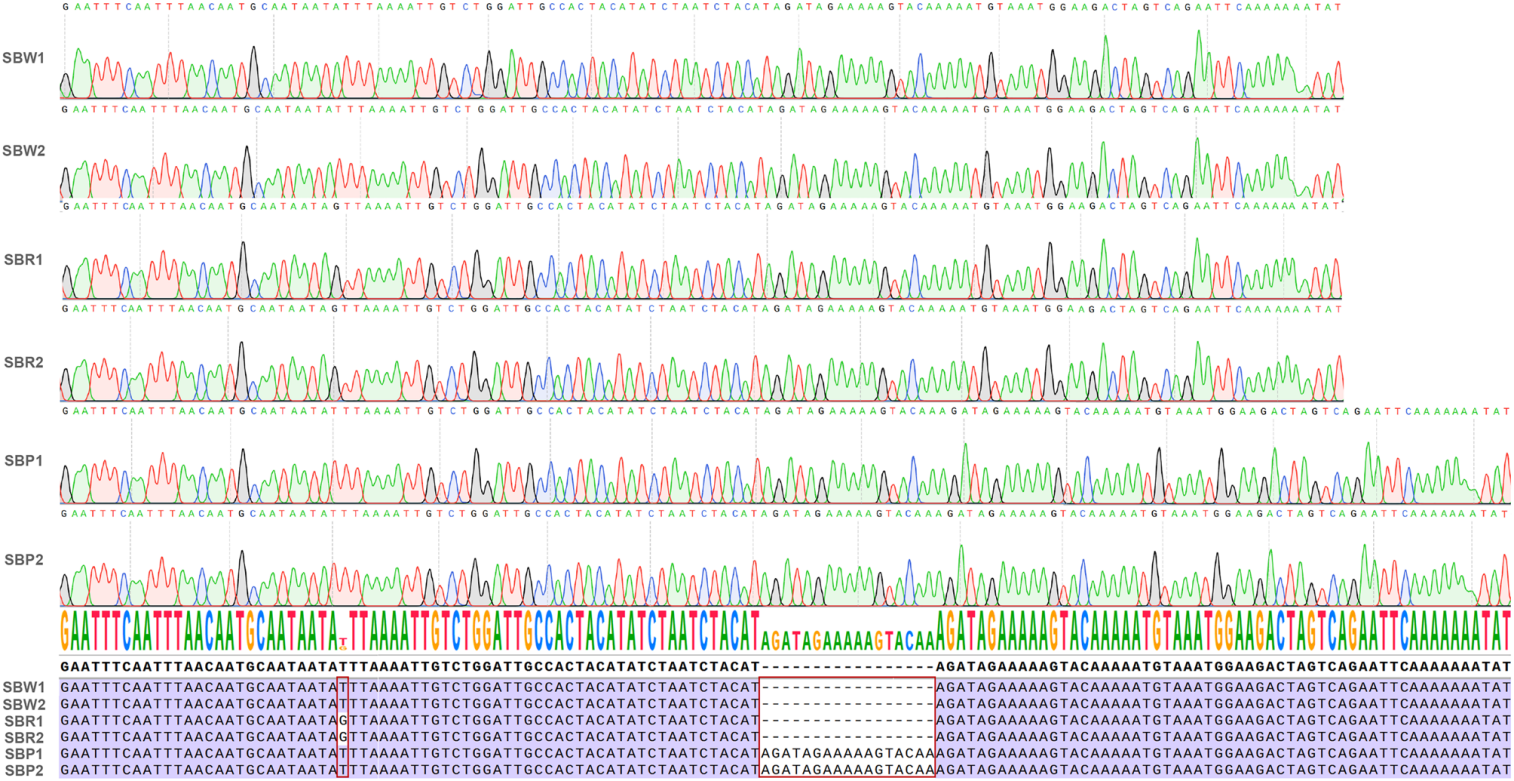
Sequencing chromatograms of the barcode regions from SBW1, SBW2, SBR1, SBR2, SBP1 and SBP2, with consensus sequence and alignment.

To discriminate the three cultivated varieties, we selected 4427–5018 bp hypervariable regions, matK-rps16, to develop a barcode in which primer sequence F (forward, 5′–3′): GAATTTCAATTTAACAATGCAATAATA and R (reverse, 5′–3′): ATATTTTTTTGAATTCTGAC. PCR amplification of total DNAs from all five medicinal species samples resulted in products having the expected size (Fig. S2). The DNA fragments were extracted from each band and then subjected to Sanger sequencing. The sequencing results were identical to the expected sequences (Fig. 8). The barcode has a specific SNP loci and one Indel loci. These two variable loci can be used to differentiate three cultivated varieties.

### Phylogenetic analysis and divergence times analysis

**Figure 9:**
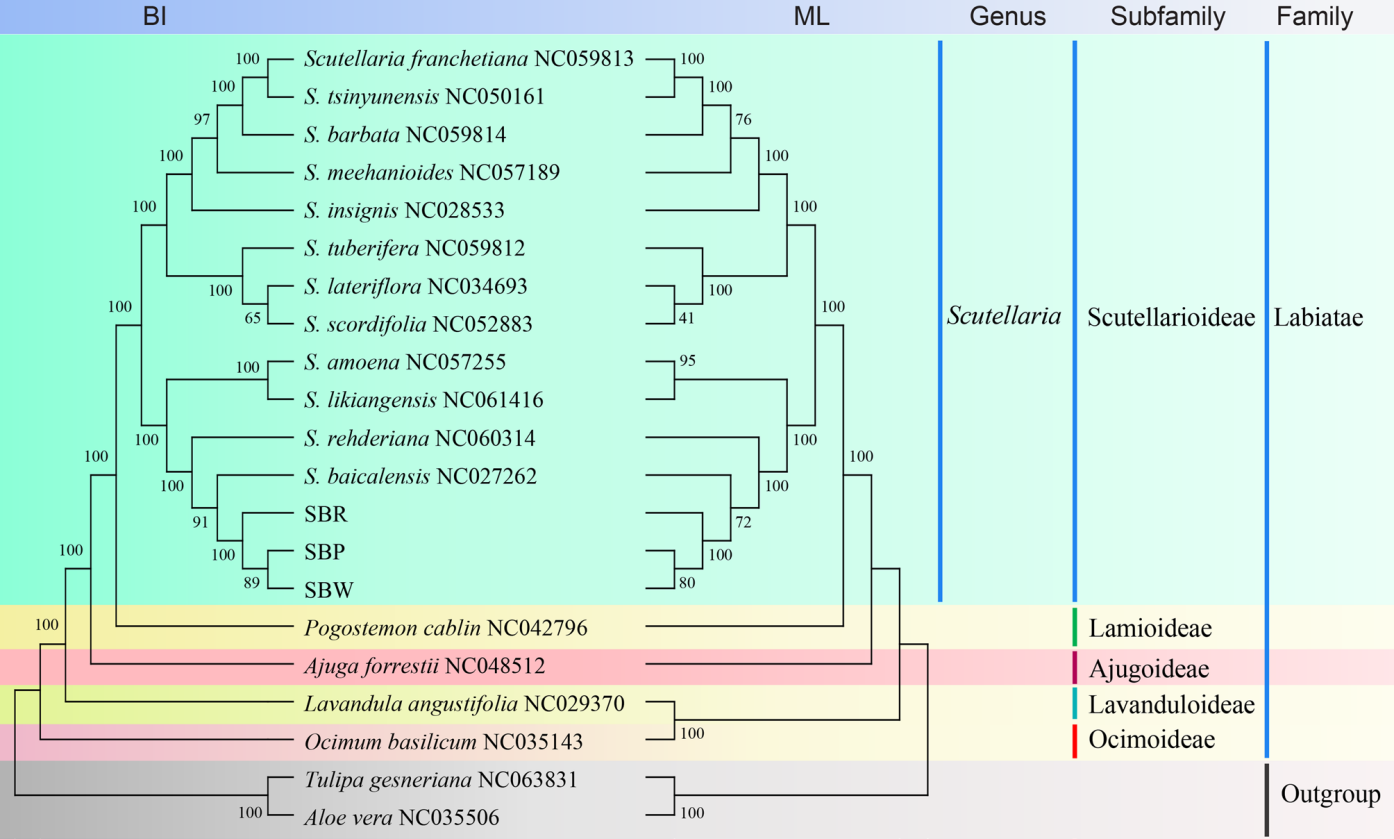
BI and ML phylogenetic tree based on 87 cp genes of the 21 species. The bootstrap support values are listed at each node.

Each subfamily in the Labiatae formed a monophyletic clade. Scutellarioideae, Lamioideae, Ajugoideae, Lavanduloideae, Ocimoideae were sister groups to each other. This result is consistent with previous genetic studies^49^. The *Scutellaria* belongs to the Scutellarioideae subfamily. Moreover, the Flora of China classifies *Scutellaria* into Subgen. *Scutellaria*, Subgen. *Anapis* and Subgen. *Scutellariopsis*. However, the results of this study do not support such a classification. The BI and ML phylogenetic trees (Fig. 9) and phylogram (Fig. S3) revealed that SBP was more closely related to SBW, in the three cultivated varieties, which was also consistent with the result of the oligonucleotide repeats analysis. In addition, three cultivated varieties together with *S. baicalensis* (NC027262). They formed a strongly supported sister relationship with *S. rehderiana* (NC060314) and clustered into one branch, and then, with *S. amoena* (NC057255) and *S likiangensis* (NC061416) cluster together. This finding was consistent with the previous studies^50^. The closely related plants may possess similar chemicals and have the same pharmacological properties. Moreover, plants are phylogenetically related to each other. Therefore, ethnobotanists have used a range of phylogenetic methods for bioprospecting^51^. According to previous research, the main pharmaceutical active ingredients of *S. baicalensis* are flavonoids, glycosides and aglycones^52,53^. Modern pharmacological studies show that the active ingredients of *S. baicalensis* have anti-bacterial, anti-tumor, anti-oxidation, anti-viral, and anti-inflammation properties^6–8^. These results provide new ideas for the exploitation of *S. baicalensis*. The cp genomes seemed to provide more solid support for the reconstruction of phylogenetic relationships among these sections.

**Figure 10:**
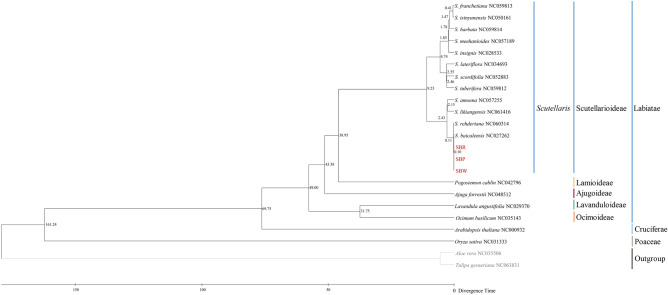
Divergence times tree obtained from a molecules clock analysis using the MEGA software. The node ages are given for each node.

The molecular clock trees were calibrated by MEGA and BEAST with fossil record data of *A. thaliana*-*O. sativa* (Figs. 10 and S4). *Ocimum basilicum* and *Lavandula angustifolia* as root species of Labiatae with a divergence time estimated at 49.00 Mya (Fig. 10). The monophyletic group of the *Scutellaris* genus diverged at about 38.95 Mya. In a previous study, the divergence time of *S. baicalensis* based on genome sequence was approximately 13.28 Mya^4^. While based on the *mat*K and *CHS* genes, the divergence time of *S. baicalensis* and *S. salviifolia* was approximately 1.37 Mya^54^. This study confirmed and traced the divergence time of *S. baicalensis* and three cultivated varieties, which occurred at 0.11 Mya and 0.10 Mya, later than previously reported. The differences in divergence time between three cultivated varieties and *S. baicalensis* are likely due to the influence of the amount of data and hybridization^55,56^.

## Conclusions

In this study, the cp genome of the three cultivated varieties of *S. baicalensis* were sequenced and assembled. A comparative analysis with other genomes was also performed. *S. baicalensis* is one of the most commonly used Chinese medicinal materials in China. The study of cp genome can provide more biological information for the sustainability of *S. baicalensis*. Overall, the three cultivated varieties of *S. baicalensis* cp genomes had similar structures and gene compositions. However, the sliding window results show significant differences among the three cultivated varieties in *mat*K-*rps*16 and *pet*A-*psb*J. Therefore, the complete cp genome could be used as a super-barcode to identify the three cultivated varieties. Moreover, we verified that the *mat*K-*rps*16 sequence can be used as a barcode for the identification of three varieties. We reconstructed a phylogenetic tree by complete cp genomes. The result indicated that *S. baicalensis* and *S. rehderiana* are closely related. The results provide new ideas for the exploitation of *S. baicalensis*. In addition, the divergence time analysis showed that the three cultivated varieties diverged at about 0.10 Mya. Overall, these results can provide species identification and biological information and contribute to the bioprospecting and improvement of ornamental value.

### Sample collection and experiment statement

All the methods including plant leaves collection and experiment were carried out in accordance with relevant national/international/legislative and institutional guidelines and regulations.

## Supplementary Information

Supplementary Figures.

Supplementary Table S1.

## Data Availability

All sequences used in this study are in the form of attachments. We have submitted this part of the data to NCBI but have not yet released it. At present, we have provided it to the journal and reviewers as an attachment and urge NCBI to release it as soon as possible. The dataset generated and or analyzed during the current study is deposited in Genbank with accession numbers: OP837955, OP837956 and OP837957.

## Abbreviations

cp: Chloroplast
NCBI: National Center for Biotechnology Information
LSC: Large single-copy
SSC: Small single-copy
IR: Inverse repeat
SSRs: Simple sequence repeats
ML: Maximum likelihood
BI: Bayesian inference

## References

[1] Zhao T (2019). *Scutellaria*
*baicalensis* Georgi. (Lamiaceae): A review of its traditional uses, botany, phytochemistry, pharmacology and toxicology. J. Pharm. Pharmacol..

[2] National Pharmacopoeia Committee, N. *Pharmacopoeia of the People’s Republic of China* (2020).

[3] Wang Z (2018). A Comprehensive review on phytochemistry, pharmacology, and flavonoid biosynthesis of *Scutellaria*
*baicalensis*. Pharm. Biol..

[4] Xu Z (2020). Comparative genome analysis of *Scutellaria*
*baicalensis* and *Scutellaria*
*barbata* reveals the evolution of active flavonoid biosynthesis. Genomics Proteomics Bioinformatics.

[5] Liao H, Ye J, Gao L, Liu Y (2021). The main bioactive compounds of *Scutellaria*
*baicalensis* Georgi. for alleviation of inflammatory cytokines: A comprehensive review. Biomed. Pharmacother..

[6] Park J, Kim R, Park E (2011). Antioxidant and Α-glucosidase inhibitory activities of different solvent extracts of skullcap (*Scutellaria*
*baicalensis*). Food Sci. Biotechnol..

[7] Wu R (2018). Baicalein targets GTPase-mediated autophagy to eliminate liver tumor-initiating stem cell-like cells resistant to mTORC1 inhibition. Hepatology.

[8] Ma Q (2018). San Wu Huangqin decoction, a Chinese herbal formula, inhibits influenza a/PR/8/34 (H1N1) virus infection in vitro and in vivo. Viruses.

[9] Chen L (2011). Synergistic activity of baicalein with ribavirin against influenza a (H1N1) virus infections in cell culture and in mice. Antivir. Res..

[10] Guo L (2013). Effects of ecological factors on secondary metabolites and inorganic elements of *Scutellaria*
*baicalensis* and analysis of Geoherblism. Sci. China Life Sci..

[11] Ye Q, Wang B, Mao J (2020). Cytokine storm in COVID-19 and treatment. J. Infect..

[12] Yu F (2014). Effects of baicalin in CD4+ CD29+ T cell subsets of ulcerative colitis patients. World J. Gastroenterol.

[13] Zhang Y (2013). Baicalein selectively induces apoptosis in activated lymphocytes and ameliorates concanavalin a-induced hepatitis in mice. PLoS ONE.

[14] Boozari M, Hosseinzadeh H (2021). Natural products for COVID-19 prevention and treatment regarding to previous coronavirus infections and novel studies. Phytother. Res..

[15] Liu H (2021). *Scutellaria*
*baicalensis* extract and baicalein inhibit replication of SARS-CoV-2 and its 3C-like protease in vitro. J. Enzym. Inhib. Med. Chem..

[16] Song J (2021). The comprehensive study on the therapeutic effects of baicalein for the treatment of COVID-19 in vivo and in vitro. Biochem. Pharmacol..

[17] Neuhaus HE, Emes MJ (2000). Nonphotosynthetic metabolism in plastids. Annu. Rev. Plant Biol..

[18] Palmer JD, Jansen RK, Michaels HJ, Chase MW, Manhart JR (1988). Chloroplast DNA variation and plant phylogeny. Ann. Mo. Bot. Gard..

[19] Henriquez CL (2020). Evolutionary dynamics of chloroplast genomes in subfamily Aroideae (Araceae). Genomics.

[20] Chen Q, Wu X, Zhang D (2019). Phylogenetic analysis of *Fritillaria*
*cirrhosa* D. Don and its closely related species based on complete chloroplast genomes. PeerJ.

[21] Zhang W (2021). DNA barcoding of *Oryza*: Conventional, specific, and super barcodes. Plant Mol. Biol..

[22] Li X (2015). Plant DNA barcoding: From gene to genome. Biol. Rev..

[23] Shi L (2019). CPGAVAS2, an integrated plastome sequence annotator and analyzer. Nucleic Acids Res..

[24] Chan PP, Lin BY, Mak AJ, Lowe TM (2021). TRNAscan-SE 2.0: Improved detection and functional classification of transfer RNA genes. Nucleic Acids Res..

[25] Zhang D (2020). PhyloSuite: An integrated and scalable desktop platform for streamlined molecular sequence data management and evolutionary phylogenetics studies. Mol. Ecol. Resour..

[26] Chen C (2020). TBtools: An integrative toolkit developed for interactive analyses of big biological data. Mol. Plant..

[27] Benson G (1999). Tandem repeats finder: A program to analyze DNA sequences. Nucleic Acids Res..

[28] Beier S, Thiel T, Münch T, Scholz U, Mascher M (2017). MISA-web: A web server for microsatellite prediction. Bioinformatics.

[29] Kurtz S (2001). REPuter: The manifold applications of repeat analysis on a genomic scale. Nucleic Acids Res..

[30] Frazer KA, Pachter L, Poliakov A, Rubin EM, Dubchak I (2004). VISTA: Computational tools for comparative genomics. Nucleic Acids Res..

[31] Amiryousefi A, Hyvönen J, Poczai P (2018). IRscope: An online program to visualize the junction sites of chloroplast genomes. Bioinformatics.

[32] Katoh K, Standley DM (2013). MAFFT multiple sequence alignment software version 7: Improvements in performance and usability. Mol. Biol. Evol..

[33] Nylander JA, Ronquist F, Huelsenbeck JP, Nieves-Aldrey J (2004). Bayesian phylogenetic analysis of combined data. Syst. Biol..

[34] Stamatakis A (2014). RAxML Version 8: A tool for phylogenetic analysis and post-analysis of large phylogenies. Bioinformatics.

[35] Kumar S, Stecher G, Li M, Knyaz C, Tamura K (2018). MEGA X: Molecular evolutionary genetics analysis across computing platforms. Mol. Biol. Evol..

[36] Liu Y (2020). Whole-genome sequencing and analysis of the Chinese herbal plant *Gelsemium*
*elegans*. Acta Pharmaceutica Sinica B..

[37] Gao Y (2018). De novo genome assembly of the red silk cotton tree (*Bombax*
*ceiba*). GigaScience..

[38] Kar, R. K. *On the Indian Origin of Ocimum (Lamiaceae): A Palynological Approach*. (1993).

[39] Bouckaert R (2014). BEAST 2: A software platform for bayesian evolutionary analysis. PLoS Comput. Biol..

[40] Rambaut A, Drummond AJ, Xie D, Baele G, Suchard MA (2018). Posterior summarization in Bayesian phylogenetics using tracer 1.7. Syst. Biol..

[41] Helfrich, P., Rieb, E., Abrami, G., Lücking, A. & Mehler, A. TreeAnnotator: Versatile visual annotation of hierarchical text relations. In *Proceedings of the Eleventh International Conference on Language Resources and Evaluation (LREC 2018)*, 2018.

[42] Guo M (2022). Plastid genome data provide new insights into the phylogeny and evolution of the genus *Epimedium*. J. Adv. Res..

[43] Wang J (2022). Comparative analysis of chloroplast genome and new insights into phylogenetic relationships of *Polygonatum* and tribe Polygonateae. Front. Plant Sci..

[44] Wang Y (2021). Chloroplast genome variation and phylogenetic relationships of *Atractylodes* species. BMC Genomics.

[45] Hübschmann T, Hess WR, Börner T (1996). Impaired splicing of the Rps 12 transcript in ribosome-deficient plastids. Plant Mol. Biol..

[46] Vogel J, Hübschmann T, Börner T, Hess WR (1997). Splicing and intron-internal RNA editing of trnK-matK transcripts in barley plastids: Support for MatK as an essential splice factor. J. Mol. Biol..

[47] Amiryousefi A, Hyvönen J, Poczai P (2018). The chloroplast genome sequence of bittersweet (*Solanum*
*dulcamara*): Plastid genome structure evolution in Solanaceae. PLoS ONE.

[48] Mehmood F, Shahzadi I, Ahmed I, Waheed MT, Mirza B (2020). Characterization of *Withania*
*somnifera* chloroplast genome and its comparison with other selected species of Solanaceae. Genomics.

[49] Jiang D (2017). The chloroplast genome sequence of *Scutellaria baicalensis* provides insight into intraspecific and interspecific chloroplast genome diversity in *Scutellaria*. Genes..

[50] Yang X (2022). Advances in pharmacology, biosynthesis, and metabolic engineering of *Scutellaria*-specialized metabolites. Crit. Rev. Biotechnol..

[51] Teixidor-Toneu I, Jordan FM, Hawkins JA (2018). Comparative phylogenetic methods and the cultural evolution of medicinal plant use. Nature Plants..

[52] Tan Y (2022). Pharmacological properties of total flavonoids in *Scutellaria*
*baicalensis* for the treatment of cardiovascular diseases. Phytomedicine.

[53] Zheng W (2021). Inhibitory effects of Coptidis Rhizoma on the intestinal absorption and metabolism of *Scutellariae*
*radix*. J. Ethnopharmacol..

[54] Chiang Y, Huang B, Liao P (2012). Diversification, biogeographic pattern, and demographic history of Taiwanese *Scutellaria* species inferred from nuclear and chloroplast DNA. PLoS ONE.

[55] Liu H (2022). Complete chloroplast genome sequence of *Triosteum*
*sinuatum*, insights into comparative chloroplast genomics, divergence time estimation and phylogenetic relationships among dipsacales. Genes.

[56] Drew BT, Sytsma KJ (2013). The South American radiation of *Lepechinia* (Lamiaceae): Phylogenetics, divergence times and evolution of dioecy. Bot. J. Linn. Soc..

