# Supplementary material for: Identification of three cultivated varieties of Scutellaria baicalensis using the complete chloroplast genome as a super-barcode
Source: Sci Rep. 2023 Apr 5;13:5602. doi: 10.1038/s41598-023-32493-9 (PMC10075158; doi:10.1038/s41598-023-32493-9)
Supplement: Supplementary file 1 — Supplementary Figures. [file 41598_2023_32493_MOESM1_ESM.doc]

Fig. S1. The coverage depth of the three cultivated varieties cp genomes. The raw sequence reads were mapped to the reference cp sequences. a) SWB; b) SWR; c) SWP. The X-axis shows the cp genomes length. The Y-axis shows the coverage depth of the mapped reads.


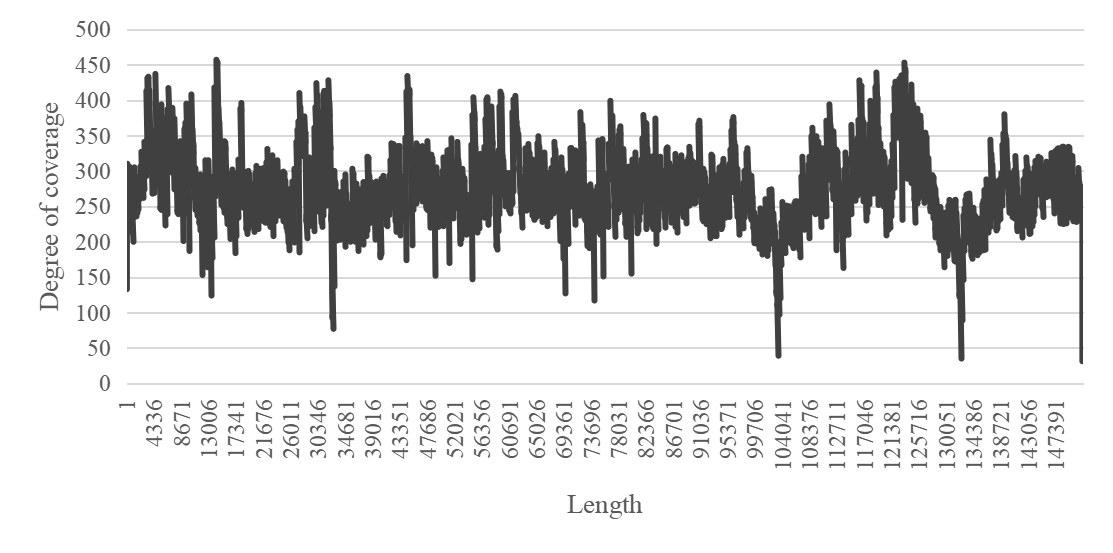

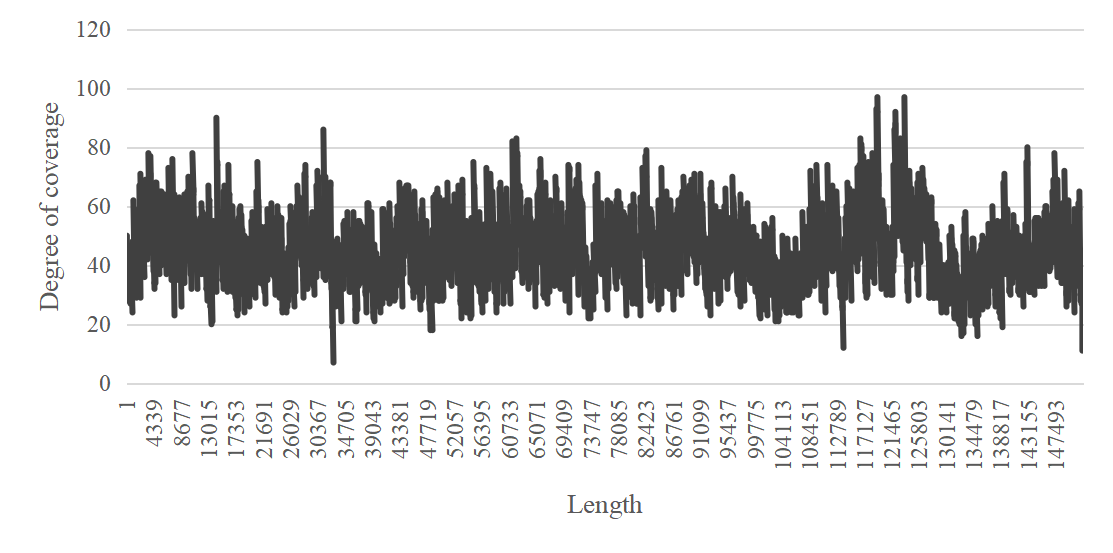

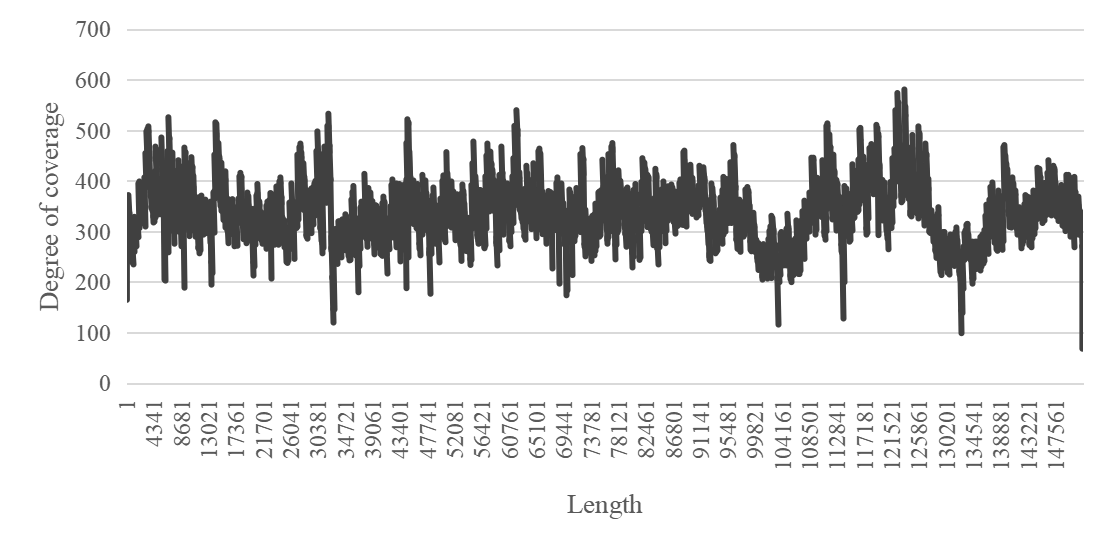


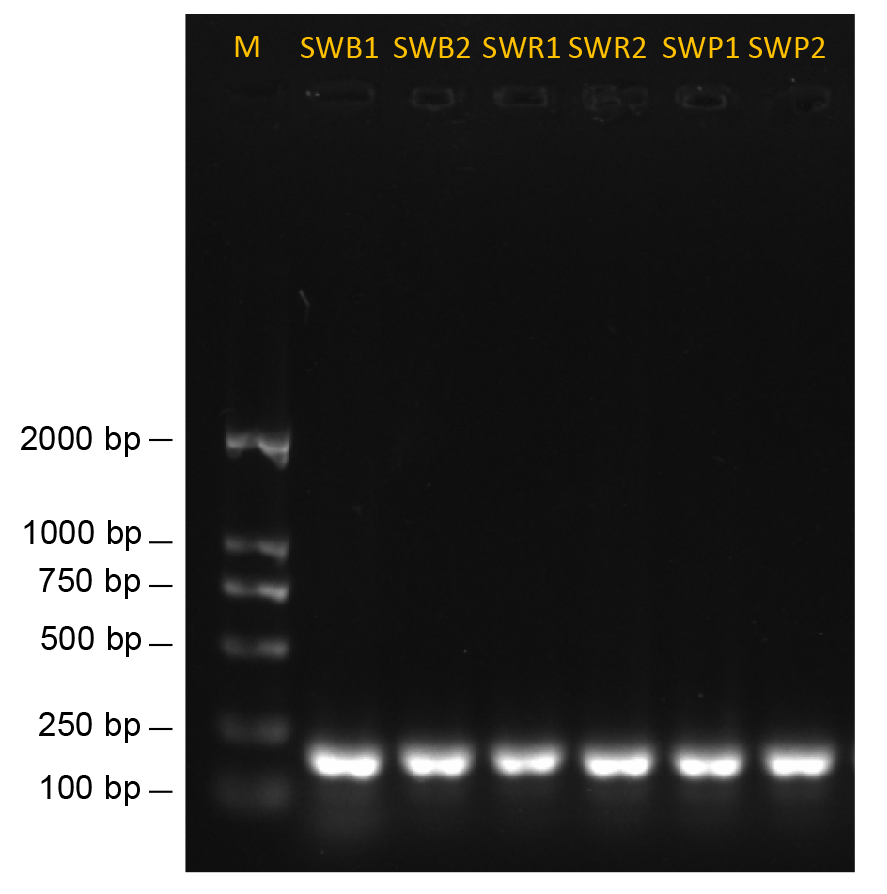


Fig. S2. The gel electrophoresis results of the PCR products. Lane M was the marker of DL 2000. The lanes from left to right corresponded to products of SBW1, SBW2, SBR1, SBR2, SBP1 and SBR2, respectively.

Fig. S3. ML phylogram tree based on 87 cp genes of the 21 species. The three cultivated varieties are highlighted in red. The bootstrap scores are shown on the corresponding branches.


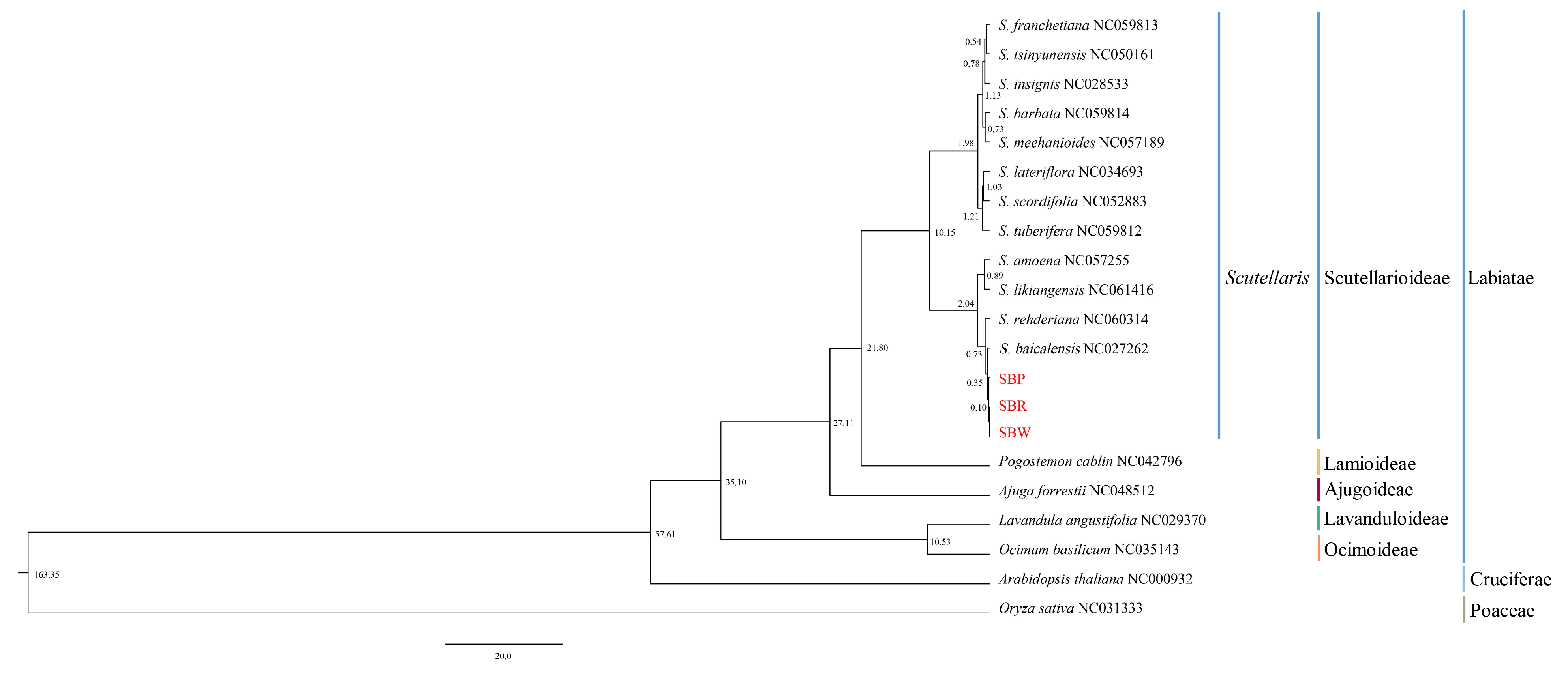


Fig. S4. Divergence times tree obtained from a molecules clock analysis using the BEAST software. The node ages are given for each node.
